# Alternative Nesting Strategies of Polistine Wasps in a Subtropical Locale

**DOI:** 10.3390/insects13010053

**Published:** 2022-01-04

**Authors:** Scott Nacko, Mark A. Hall, Gregg Henderson

**Affiliations:** 1 Louisiana State University Agricultural Center, Department of Entomology, Baton Rouge, LA 70803, USA; GRHenderson@agcenter.lsu.edu; 2 Hawkesbury Institute for the Environment, Western Sydney University, Richmond, NSW 2753, Australia; Mark.Hall@westernsydney.edu.au

**Keywords:** eusociality, voltinism, *Polistes*, *Mischocyttarus*, phenology

## Abstract

**Simple Summary:**

Paper wasps are eusocial insects which serve as excellent models for studying the evolution of sociality. In this study we provide a new and unique view of the nesting biology of social wasps by comparing and contrasting the phenology of species in two genera, *Polistes* and *Mischocyttarus*, in a context that sheds light on the life history and evolution of this group. Over the course of one year in subtropical Baton Rouge, USA, we found two nesting strategies, with *Polistes* having a second nesting cycle characterized by an abbreviated colony duration and smaller nests, and *Mischocyttarus mexicanus* having one nesting cycle characterized by long colony duration and few late season nests. Our results highlight phenological differences in the life history of an assemblage of social wasps in a subtropical locale, and support previous foundational works concerning the ancestral origins of the Polistinae.

**Abstract:**

Phylogenetic studies suggest that historically all paper wasps (Vespidae: Polistinae) in North America have tropical origins, but some species have adapted to survive temperate conditions. Subtropical climates, which are intermediate between temperate and tropical, allow a unique opportunity to study ancestral traits which can be retained or lost within populations, and ultimately elucidate the process of social wasp evolution. We investigated the phenology of paper wasps at study sites in subtropical Baton Rouge, USA, through nest searching and monitoring of nest parameters throughout the warm season (March–October). Across the year, two periods of nest initiation occurred: from March–May (early season nests, i.e., before the summer solstice), and from July–September (late season nests, after the solstice). We observed 240 *Polistes* nests from six species, of which 50.8% were initiated in early season and 49.2% in late season. In contrast, *Mischocyttarus mexicanus* rarely built late season nests and had longer early season colony duration than *Polistes bellicosus* and *P. dorsalis,* which built more nests in the late season than early. Across all species, late season nests had significantly shorter colony duration (~87.6 days) than early season nests (~166 days), and only *P. bellicosus* had fewer adults at peak population in late season nests than in early season nests. Results indicate both a bivoltine colony cycle in *Polistes* of subtropical climates, as well as differences in nesting strategies between genera.

## 1. Introduction

In North America, paper wasps (Hymenoptera: Vespidae: Polistinae) exhibit strong phenological traits [1]. Phenology, or the study of adaptations which align the biological activities of a species with environmental cycles [2], is often used to investigate genetic expression, effects of climate change, and life-history evolution [3,4,5]. For instance, in insects living in temperate zones, a period of diapause allows them to survive the cold season and exploit the warm season for growth and reproduction [6], but a lengthened growing season presents them with a tradeoff between optimal responses: to reach reproductive maturity earlier in the season or to grow larger before reproducing [4]. In the case of social insects such as paper wasps, reproductive fitness is measured by the total number of reproductive individuals produced by a colony at the end of a season [7], thus the tradeoff at a colony level is either to have two nesting cycles per season or to grow larger colonies. Variations in phenological traits can result from environment x gene interactions, and have been documented in ants and crickets [8,9].

Paper wasps are eusocial (worker and queen containing) insects comprised of four tribes and >1000 described species worldwide, with the majority of species diversity occurring in tropical and subtropical climates [10]. There are two major life histories within the Polistinae, with swarm founding species displaying long (>1 year) colony duration and no phase without workers (highly eusocial), and solitary founding species displaying short colony duration (<1 year) and a foundation phase without workers (primitively eusocial) [10]. Genera such as *Polistes* and *Mischocyttarus* have a largely solitary founding life history, where nests are founded by one or multiple queens which exert dominance through aggressive interactions [10,11,12].

The number of generations produced per year, or voltinism [13], is dependent upon a combination of environmental cues and genotypic expression [8]. Photoperiod, temperature, and food availability all mediate the phenological activities of temperate zone paper wasps [2,14] and result in synchronous nesting—all nests across a local region being in similar developmental phase [1,15]. The nesting cycle of paper wasps in temperate zones is typically univoltine, with one cycle occurring each year [1,15]. In North America, the foundation of nests and production of workers occurs when day length increases in Spring (April or May), and the production of reproductive females (gynes) and males, along with colony decline/abandonment, occurs when day length decreases (July–October) [1]. Gynes emerge in a physiological state of reproductive diapause [16] and summers are not long enough for to colony cycles [17] in temperate North America. In tropical zones the nesting cycle is multivoltine and annual synchrony lost; nests undergo the same developmental stages and last approximately the same length of time as elsewhere, but lack correlation between developmental phase and time of year [1,18]. Those in subtropical zones (−3 °C < T_min_ < +18 °C, T_max_ ≥ +22 °C) [19] generally demonstrate longer nesting periods (6 to 8 months) than temperate zones but still display annual synchrony and univoltinism [20].

Whether ancestral polistines first arose in a temperate or tropical climate has long been a topic of interest. Recent evidence strongly supports tropical Southeast Asia as a place of origin [10]. Some authors have argued that *Polistes* evolved from solitary bivoltine ancestors in a seasonal climate, with the worker (G1) and gyne (G2) phenotypes corresponding with the two-generational life history in the solitary ancestor [16]. In this theory, both the worker and gyne phenotype are reproductive, with the suppression of reproduction in workers being only context dependent. Indeed, in the primitively eusocial *Polistes* and *Mischocyttarus*, the lack of morphological differentiation between castes leaves open the possibility for direct reproduction in workers [21,22].

The southern United States is a unique area to study the phenology of paper wasps: two genera occur in sympatry at the southern extent of temperate species distributions and the northern extent of tropical species distributions. *Polistes* and *Mischocyttarus* are speciose genera, the former occurring on all six habitable continents [23] while the latter is restricted to the neotropics, with only three species reaching the temperate climates of North America [13]. By comparing and contrasting the phenology of a polistine species assemblage in a subtropical climate, we aim to explore how these social insects respond to a lengthened warm season and discuss potential implications for the evolution of sociality in this group. We pose three main questions:

(1) Do polistine wasps in a subtropical locale initiate nests in both early and late season?
(2) Does colony duration and peak number of adults, pupae (capped cells), and cells differ between early and late season?
(3) Does the proportion of single foundress and multi foundress nests differ between early and late season among species?

## 2. Materials and Methods

### 2.1. Study Area and Design

We investigated nests at four sites that were located across a 12 square mile area in Baton Rouge and St. Gabriel, Louisiana, USA: Louisiana State University main campus (30°24′52.5″ N 91°10′34.8″ W), LSU Burden Museum & Gardens (30°24′31.1″ N 91°06′20.0″ W), LSU AG Center (30°16′19.5″ N 91°05′59.2″ W), and Bluebonnet Swamp Nature Center (30°22′08.5″ N 91°06′18.6″ W). Köppen climate classification indicates these areas have a humid subtropical climate with an average annual temperature of 20 °C and annual rainfall of 1526 mm [24]. To locate as many nests for study as possible in both seasons, weekly nest searches began during February 2016 and continued simultaneously during data collection of previously discovered nests until November 2016. We checked for wasp nesting activity in January–February and November–December, however no nesting activity was observed, possibly due to a period of physiological diapause. As air temperature is a major factor affecting wasp activity [25], and *Polistes* generally begin flying at 18 °C [26], nest searching was conducted on warm and sunny days when the temperature was ≥18 °C, with search durations of ~4 h each day.

### 2.2. Data Collection

Nest searches and observations were made of polistine wasps in two genera: *Polistes* and *Mischocyttarus.* Nest searches were conducted by visual inspection of typical nesting structure for these species: palm fronds, picnic shelters, eaves of buildings, crevices, and low shrub vegetation. Wasps were also occasionally observed flying directly to a specific nest location. Upon discovery, each nest was assigned a unique identification number, and its location and species were recorded. Species identifications were made by SN using the Vespid Identification Atlas [27] and verified by Dr. Matthias Buck at the Royal Alberta Museum (Edmonton, Canada). Voucher specimens were collected (one female from each commonly encountered species) and deposited at the Louisiana State Arthropod Museum. Nests were typically discovered containing eggs or small larvae, making it possible to estimate the month in which they were initiated. When a nest contained larvae upon discovery, it was estimated to have been initiated at least ten days prior to discovery, as this is the minimum amount of time required for egg development in *Polistes* spp. of the southern USA [28]. As photoperiod is an important cue regulating the activities of temperate polistines [1,14], a nest was considered to be an “early season” nest if initiation had occurred while daylight was increasing before the summer solstice (20 June) and a “late season” nest if initiation occurred after the solstice while daylight was decreasing. Nests which were already well developed (in the post-emergence phase) but discovered after July were excluded from our study, as it was not possible to determine in which season they were initiated. Of the study nests, a nest was considered to be “multiple foundress” if more than one female was consistently recorded (≥2) on the nest during the pre-emergence phase, “single foundress” if only one female was recorded during the pre-emergence phase, or “unknown” if the nest was at the post-emergence phase when discovered.

Weekly visits were made to every nest from the time of its discovery until nest abandonment to record number of adults, cells, and pupal caps present. Additional nests were discovered during visits to known nests, so weekly nest searches were continued throughout the summer. A Canon PC1339 digital camera (12 × zoom) was used to photograph the comb face of each nest during each visit, allowing us to quantify data parameters from well-lit, still photographs while avoiding stings and disruptions to colony activities. When a nest was situated such that viewing of the comb face was not possible, it was not included in the study of adults, pupal caps, cells or duration. As eggs and larvae require adult care to survive [1], colony duration was defined as the length of time (from discovery date) during which brood (live eggs and/or larvae) and adult females were present in the nest. When a nest already contained larvae upon discovery, 10 days were added to its duration, as this is the typical developmental time for eggs in *Polistes* spp. of the southern USA [28]. Similarly, if a nest was already in the post-emergence phase (four instances) when discovered, 46 days were added to its duration, as this is the typical developmental time from egg to adult [28]. Nests which failed before reaching natural abandonment were not included in the analysis of colony duration. A nest was considered naturally abandoned once it contained no brood and no adult females. Failure was defined as premature nest abandonment, absence, or human caused fatality. When recording the number of adults, those which were completely out of visible range (i.e., hidden on top of the nest) were not recorded. Behavioral observations, such as dominance interactions or movements between nests, were also noted while visiting each nest. “Peak adults” for a given nest was defined as the highest number of adults observed on that nest over the full course of observations, as is similar to “peak pupal caps” being the highest number of silken covered cells (or pupal caps) over the course of observations. Cell number was defined as the total number of cells, with or without cap remnants, per nest at the conclusion of observations.

### 2.3. Statistical Analyses

We carried out a series of analyses to examine: (a) periods of nest initiation, (b) colony duration and nest parameters, and (c) polistine nesting behavior. All statistical analyses were performed in R version 3.6.1 [29], using the packages: *stats* (v.3.6.1) [30], *emmeans* (v.1.4.5) [31], and *lme4* (1.1-24) [32]. Two species, *P. exclamans* and *P. annularis,* did not have sufficient numbers (>2) of surviving nests to include in most analysis, however *P. exclamans* was used in models of nest foundresses. Likewise, surviving early season nests of *P. fuscatus* (*n* = 1) were not sufficient to include in colony duration analysis.

First, we determined if there was a difference in the number of nests between early and late seasons for each species, adopting a generalised linear model (GLM) approach. To account for differences in variances in relation to levels of the categorical variable ‘season’ and to help normalise residuals [33], we used generalised least squares models (GLS) to include a variance function, with a qqnorm plot to examine the residual fit (Supplementary Material Figure S1). Second, we tested if there was a correlation between the number of nests discovered each month (pooled across all species) with the average monthly photoperiod (hrs:mins) per day [34], using a Spearman’s test with the function *cor.test*. Third, we determined if there was a difference in the number of days devoted to brood rearing between nesting seasons for five wasp species. We began by pooling all species to test the overall effect of season, again using a GLS model with a qqnorm plot to examine residuals (Supplementary Material Figure S2). Duration (number of days devoted to brood rearing) was set as the response variable (continuous) and season (early or late season; categorical) set as a fixed effect. We then ran individual GLS models for each of four wasp species (*P. bellicosus*, *P. dorsalis*, *P. metricus* and *M. mexicanus*) to determine if there were differences in nest duration between seasons for each. Post hoc analyses of estimated marginal means (EMM) were performed to compare species responses to seasonal nesting duration based on multiple pairwise comparisons, with a Bonferroni correction added to GLS models to account for unequal variance.

Then, we investigated differences in the abundance of adults, caps and cells at nests for five wasp species (*P. bellicosus*, *P. dorsalis*, *P. fuscatus*, *P. metricus* and *M. mexicanus*) between seasons. For each of these measures, we individually modelled the relationship of each species with season (categorical variable representing one of the two nesting periods described above). We again used GLS models to include a variance function, and examined the residuals with a qqnorm plot (Supplementary Material Figure S3). Finally, we used a generalised linear mixed model (GLMM), assuming a binomial distribution and logit link, to investigate if there were differences in the proportion of nests that had single or multiple foundresses between seasons. We used the proportion of nests from the total for each species recorded as having either single, multiple or unknown foundresses as the response variable, and season (categorical) and number of foundresses (categorical) as fixed effects. We also included an interaction term between the two fixed effects and a random effect of observations.

## 3. Results

### 3.1. Species Composition and Periods of Nest Initiation

**Figure 1:**
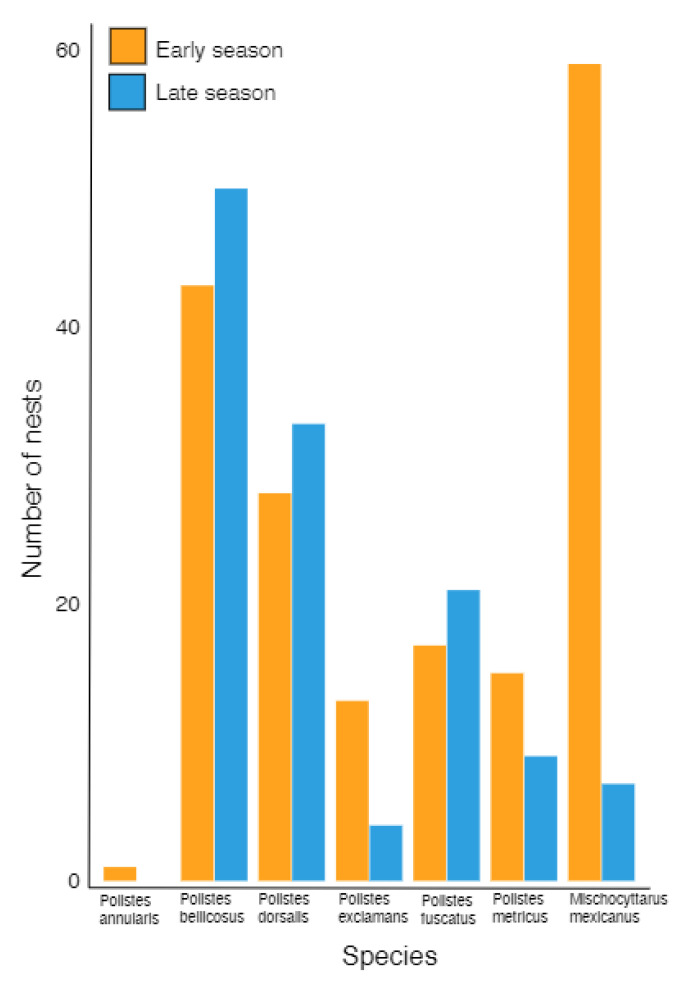
Number of nests from seven polistine species observed in early and late season during 2016.

**Figure 2:**
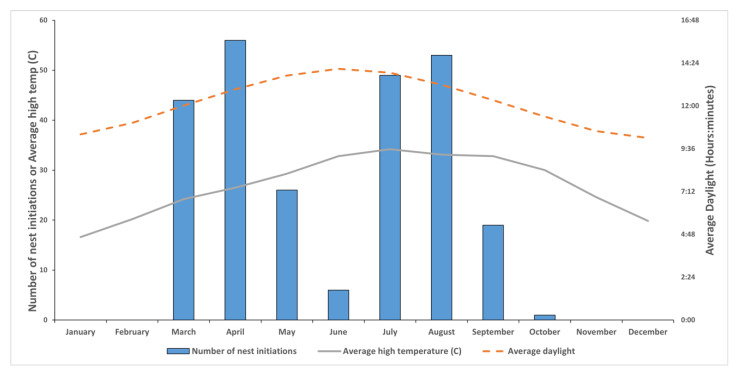
Number of polistine nest initiations per month (blue bars), average monthly high temperature (°C) (solid line), and average daylight per month (hrs:mins) (dotted line) during 2016 in Baton Rouge.

A total of 300 active paper wasp nests were located between March and October 2016; although wasps were noted flying in February, the first recorded nest initiation of the year occurred on 3 March (*P. fuscatus*), with the last recorded initiation on 4 October (also *P. fuscatus*). No nests were initiated in January–February or November–December. Of the total nests, 176 were early season nests while 124 were late season. 43% (*n* = 74) of all early season nests failed before July. The species most often observed on early nests were *Mischocyttarus mexicanus* (*n* = 54) and *Polistes bellicosus* (*n* = 36), followed by *P. dorsalis* (*n* = 30), *P. fuscatus* (*n* = 17), *P. metricus* (*n* = 14), *P. exclamans* (*n* = 12), and *P. annularis* (*n* = 1) (Figure 1). In comparison on late season nests, *P. bellicosus* (*n* = 50) and *P. dorsalis* (*n* = 31) were the two most often observed, followed by *P. fuscatus* (*n* = 19), *P. metricus* (*n* = 8), *M. mexicanus* (*n* = 6), and *P. exclamans* (*n* = 3) (Figure 1). There were a total of 19 *Polistes* nests across both seasons (12 in early, 7 in late season) where species could not be identified. Examining only *Polistes* spp. (i.e., excluding *M. mexicanus*), 50.8% (*n* = 122) of nests were initiated in the early season and 49.2% (*n* = 118) in the late season. Early season initiations began in March and reached a peak in April (*n* = 56) when the average photoperiod length was 12 h 56 min [34] and average high temperature was 26.5 °C [35], before declining in May (*n* = 26) and reaching a low point in June (*n* = 6) (Figure 2). A similar pattern was observed with late season initiations beginning in July (*n* = 49) and reaching a peak in August (*n* = 53) when the average photoperiod length was 13 h 11 min and average high temperature was 33.1 °C, before declining in September (*n* = 19) and reaching a low point in October (*n* = 1). The period in which there were no initiations (January–February and November–December 2016) had average monthly photoperiod that varied from 10 h 12 min to 11 h 03 min and average monthly high temperature from 16.6 to 24.6 °C (Figure 2). No correlation was found between photoperiod length and the number of nest initiations (t = 0.26, *p* = 0.80; Supplementary Material Figure S4).

### 3.2. Colony Duration and Nest Parameters

**Figure 3:**
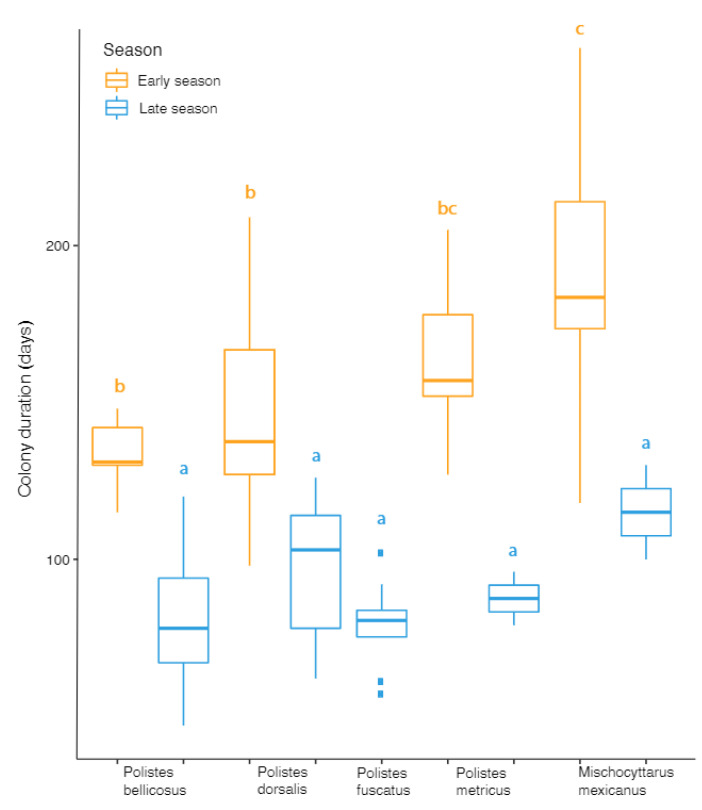
Colony duration (number of days devoted to brood rearing) for five polistine species.in early and late season nests. Lettering above shows differences based on multiple pairwise comparisons among species across each season.

For all species combined, we found a significant effect of season on colony duration, with late season nests (mean of 87.6 ± 3.8 days) having a shorter duration than early nests (166 ± 6.9 days; t = −9.84, *p* < 0.001). Each of the four species able to be compared had a shorter late season duration than early (*P. bellicosus*: t = −5.64, *p* < 0.001; *P. dorsalis*: t = −3.33, *p* < 0.01; *P. metricus*: t = −4.88, *p* < 0.01; *M. mexicanus*: t = −4.11, *p* < 0.001; Figure 3). No significant difference in colony duration was observed between species during the late season, however in early season nests, *M. mexicanus* had significantly longer colony duration than *P. bellicosus* or *P. dorsalis*. (Figure 3; Supplementary Materials Table S1). *P. metricus* early season colony duration was not different to any other species (Figure 3; Supplementary Materials Table S1).

**Figure 4:**
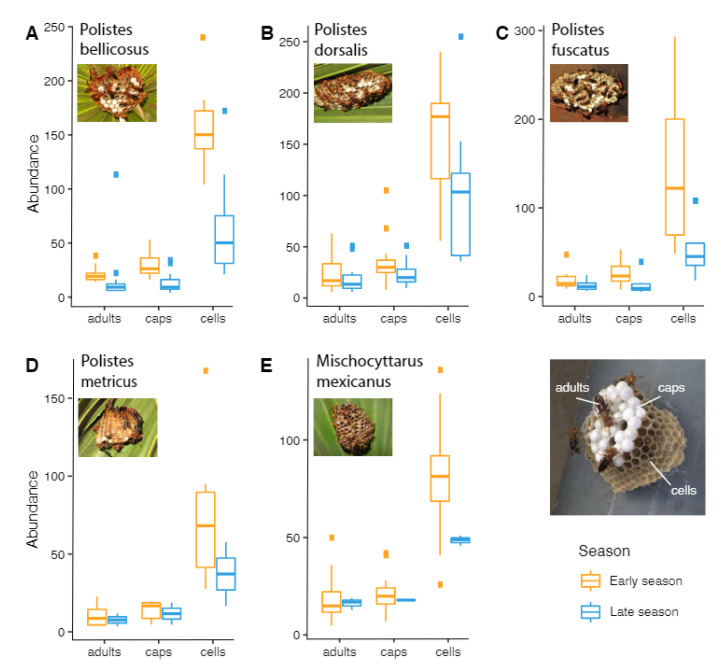
Peak number of adults, pupal caps, and cells in early and late season nests of each of five polistine species: *Polistes bellicosus* (**A**), *P. dorsalis* (**B**), *P. fuscatus* (**C**), *P. metricus* (**D**) and *Mischocyttarus mexicanus* (**E**).

The peak number of adults was significantly greater in early than in late season nests for one species, *P. bellicosus* (t = 4.81, *p* < 0.001) (Figure 4; Supplementary Materials Table S2). The peak number of pupal caps was significantly greater in early than in late season nests of *P. bellicosus* (t = 4.48, *p* < 0.001), *P. fuscatus* (t = 2.48, *p* = 0.02), and *M. mexicanus* (t = 5.28, *p* < 0.001; Figure 4; Supplementary Materials Table S2). Likewise, the number of cells per nest was significantly greater in early than in late season nests for *P. bellicosus* (t = 6.84, *p* < 0.001)*, P. dorsalis* (t = 2.27, *p* = 0.03)*, P. fuscatus* (t = 3.02, *p* = 0.01)*,* and *M. mexicanus* (t = 5.28, *p* < 0.001; Figure 4; Supplementary Materials Table S2). *P. metricus* showed no difference in any nest parameters between seasons (Supplementary Materials Table S2).

### 3.3. Polistine Nesting Behavior

**Figure 5:**
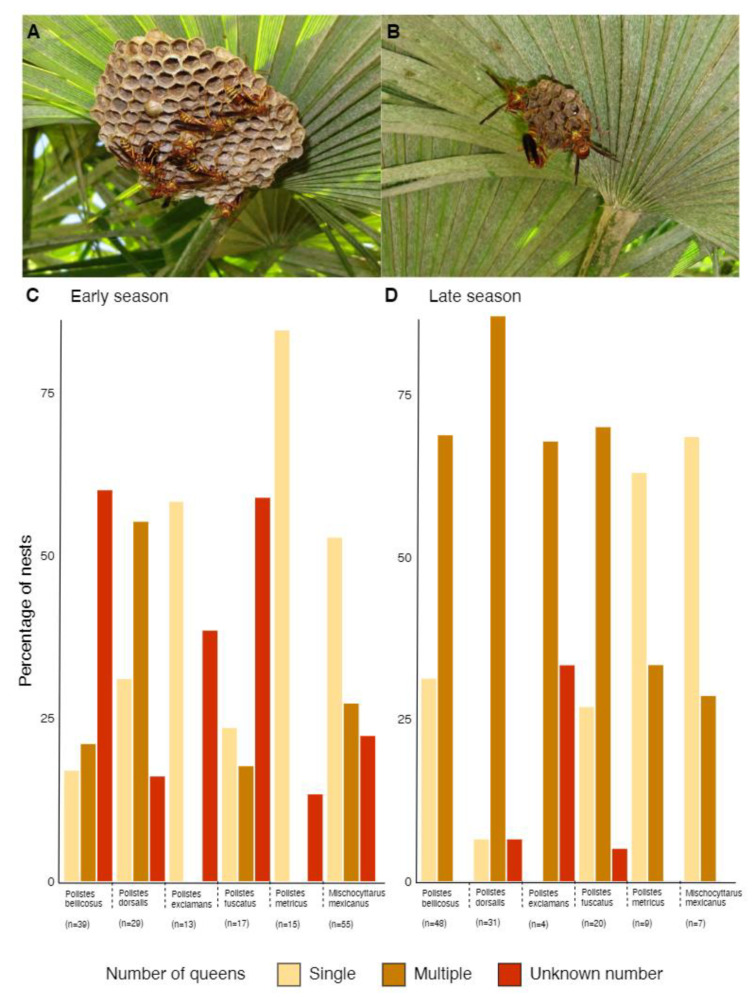
An early season nest of *P. bellicosus* on 16 August which had naturally declined (**A**) and an associated late season nest of *P. bellicosus* on 28 August (**B**). Female transfer was noted between the two nests. Percentage of single-foundress, multi-foundress and unknown number of foundress nests for each species in early (**C**) and late season (**D**).

We noted female transfer between seven separate late season nests; three in those of *P. bellicosus* and four in *P. dorsalis*. A multi-foundress *P. bellicosus* nest initiated in March under a palmetto leaf ceased brood production in late July and by 16 August, contained only 5 females, 3 males and no brood (Figure 5A). On that same day, a *P. bellicosus* nest was initiated by three females on a leaf directly below the leaf upon which the aforementioned was attached, and one female was observed flying from the old nest to the new nest on 28 August (Figure 5B). In another instance a foundress from a multi-foundress *P. dorsalis* nest initiated in late August was seen flying to a mature early season *P. dorsalis* nest just 114 cm away and actively soliciting food for ~60 s before returning to the late season nest. In this case the mature early season nest was still actively rearing brood.

There was no difference in the proportion of nests having either a single or multiple foundresses or between seasons (pooled for all species) when compared separately, however there was an interacting effect of season and number of foundresses (Supplementary Materials Table S3). There was a greater proportion of late season nests with multiple foundresses, and fewer unknown nests in the late season (Figure 5C,D). Two species (*P. exclamans* and *P. metricus*) had exclusively single foundresses (or unknown foundress number) nests during the early season (Figure 5C).

## 4. Discussion

We found evidence of two nesting periods occurring in one season across multiple *Polistes* spp. in our subtropical locale, indicating that North American ancestral *Polistes* likely had a bivoltine colony cycle as they expanded northward from the tropics, eventually forgoing the second nesting phase for a univoltine cycle in temperate zones. We also found evidence to support a fundamental difference between the nesting strategies of *Polistes* spp. and *Mischocyttarus mexicanus* in subtropical environments. While nearly equal proportions of *Polistes* nests were initiated during early and late season, with some species even having a greater number of nests in the late season, *M. mexicanus* nests were initiated almost exclusively during the early season. Late season *Polistes* nests had an abbreviated colony duration and higher proportion of multiple-foundress associations.

In our study, 58% of nests were initiated before the summer solstice in June, however 43% of these failed before July. Predation by birds is often a leading cause of failure in *Polistes* nests [17] and is likely the case here. Wasps surviving from failed nests must then either join an existing nest or initiate a new nest, possibly into the late season. Two possible scenarios could explain nest initiations of subtropical *Polistes* in July and August, including the failure of colonies early in the season. Strassmann hypothesized that satellite nests of *P. exclamans* in North America were adaptive to a high predation rate and long summer, and were built regardless of the success of the main nest [17]. In fact, 51.6% of all nests in a population of *P. exclamans* lost their queen before July, with knockdown of nests by birds being a major cause of nest mortality [17]. In another study on a suburban population of *P. fuscatus,* 34% of all nests failed to produce at least one brood [36]. However, 37% of our nests that failed before July were *M. mexicanus*, a species which rarely built late season nests. This discrepancy does not lend itself to the hypothesis of failure as the leading cause of late season nests for these sub-tropical polistines.

Reproductive plasticity is a more likely explanation for the second nesting cycle we observed in *Polistes*. It is known that “worker” or G1 females of some primitively eusocial social wasps have three equally available options for reproduction: to leave their natal nest and initiate their own either alone or in a group, to stay at the natal nest as a worker, or to stay at the natal nest and succeed the existing queen [21,22,37,38]. Early females of *P. fuscatus* are known to leave their natal nest to become foundresses the following spring [39]. Additionally, satellite nests of *P. fuscatus* are thought to result from an alternative reproductive behavior observed in polydomous colonies (those containing multiple separate combs), where workers had been pre-conditioned to tend multiple combs [36]. In such cases, the queen was not able to maintain reproductive dominance when combs were spread out, allowing for the reproductive behavior of workers. Although no polydomous early season nests were observed in our study, reproductive plasticity is a reasonable explanation behind the late season nesting period observed here. We believe the bivoltine nesting pattern we observed is due to such reproductive options, and ultimately reflects the evolutionary pathway of subtropical and temperate species from bivoltine or multivoltine tropical ancestors. Tropical environments often have a seasonal wet and dry cycle, and many tropical insects, including *Polistes versicolor* [40] and *P. canadensis* [41], are known to possess the capacity for dormancy or diapause, allowing them to reproduce at an advantageous time during the cycle [42]. Possessing a pre-existing mechanism for diapause makes a transition from tropical to temperate zones possible for some insects (i.e., Coleoptera, Diptera) [42] and such was likely the case for ancestral polistines which colonized North America. In our subtropical population, we observed a four-month period of reproductive inactivity from November–February, possibly representing physiological diapause.

Despite the warm season not being long enough for two colony cycles of *P. exclamans* in Austin, Texas USA [17], we found that a similar climate in the neighboring state of Louisiana (0.4 °C cooler on average) provided sufficient time for two colony cycles in *P. bellicosus, P. dorsalis, P. fuscatus* and *P. metricus*, albeit with an abbreviated second cycle. The second nesting cycle was characterized by short colony duration, smaller nests (fewer cells), and high occurrence of multi-foundress associations, perhaps indicative that it either lacks or has a shortened post-emergence phase. *P. bellicosus* was the only species in which the number of cells, caps and adults were all significantly fewer in late season compared to early season nests, and *P. metricus* was the only species in which none of these parameters differed. The lack of variation in *P. metricus* may be attributed to its naturally small colony size [43]. It is logical that fewer cells would lead to fewer pupal caps and thus fewer adults. We saw that, at least in *P. bellicosus, P. fuscatus* and *M. mexicanus*, fewer cells were associated with fewer pupal caps. However, peak adults were only lesser in *P. bellicosus*. The fluctuation of adult numbers as they disperse and return throughout the cycle complicates a measure of total adults produced on a nest, but our data suggest that founders of late season nests raise enough offspring to maturity to be advantageous to them.

Interestingly, our study shared a similar observation of female transfer as has been reported in the tropical *P. canadensis*. New nests of *P. canadensis* are often associated with declining mature nests, evidenced by observations of queens on new nests that were previously observed on declining nests [1]. In these cases, food solicitation and trophallaxis were noted each time the female visited the declining nest. Our observations were strikingly similar in the case of one such *P. bellicosus* nest which naturally declined and ceased brood rearing in August, after which a female was noted transferring between it and a newly initiated nest close by (Figure 4b and Figure 5a). These observations are also similar to those of satellite nests of *P. exclamans* in which satellite nests were initiated within close proximity of “main nests” by either the queen or workers, and transfer of workers between the satellite and main nest occurred until eclosion of the first brood on the satellite [17].

Nesting patterns differed between *Mischocyttarus mexicanus* and the *Polistes* spp. we observed. *M. mexicanus* was the most frequently observed wasp during the early season but comprised only 5% of late season nest initiations. This was contrary to what we expected due to its known plasticity in nesting biology—year-round nesting in Cuba, the Bahamas, Puerto Rico, and south Florida [44,45] but short diapause period in Georgia [46]. Our data demonstrate the colony duration (average of 189 days) of early season *M. mexicanus* nests was significantly longer than that of early season *P. bellicosus* and *P. dorsalis* nests, the two species most frequently observed on late season nests. This colony duration is similar to that reported for both tropical and temperate species of *Mischocyttarus* [45,47,48,49]. A strategy known as “short-term monogyny”, which favor females who remain at their natal nest to succeed the queen, has been previously noted in other species of *Mischocyttarus* and may in fact be characteristic of the genus [11,47,49]. In short-term monogyny, the reproductive dominance of the egg laying female lasts for a shorter period than the egg-adult development time, such that the nest has a successive series of reproductive egg-layers and is, in essence, polygynous [11]. Though we did not individually mark and track the foundresses in our study, short-term monogyny is a likely explanation for the longer duration of early season *M. mexicanus* nests that we observed and may ultimately reflect a differing of environments within which *Polistes* and *Mischocyttarus* arose.

## 5. Conclusions

Our findings demonstrate a new and unique view of the life histories of polistine wasps in the Southern USA. By studying the phenology of the wasp nesting cycle in a subtropical climate, we found a tradeoff in nesting strategies, with *Polistes* having a second nesting cycle characterized by an abbreviated colony duration and smaller nests, and *Mischocyttarus mexicanus* having one nesting cycle characterized by long colony duration and few late season nests. Both strategies are advantageous to G1 females of the worker phenotype, allowing the opportunity for direct reproduction. Our data support the theory of tropical ancestral origins of the Polistinae in North America.

## Data Availability

The data presented in this study are available in Supplementary Table S4 and Supplementary Table S5.

## Supplementary Materials

The following are available online at https://www.mdpi.com/article/10.3390/insects13010053/s1, Figure S1: Nest number GLS residuals, Figure S2: Duration GLS residuals, Figure S3: Colony parameters GLS residuals, Figure S4: Day length correlation, Table S1: Duration pairwise comparisons, Table S2: Nest parameter model significance, Table S3: Foundress model significance, Table S4: Early season raw data, Table S5: Late season raw data.
