# Supplementary material for: Alternative Nesting Strategies of Polistine Wasps in a Subtropical Locale
Source: Insects. 2022 Jan 4;13(1):53. doi: 10.3390/insects13010053 (PMC8777775; doi:10.3390/insects13010053)
Supplement: Supplementary file 1 [file insects-13-00053-s001.zip › insects-1520927-supplementary.pdf]

Supplementary materials for: *Alternative nesting strategies of polistine wasps in a sub-tropical region***.** Scott Nacko, Mark A. Hall & Gregg Henderson.

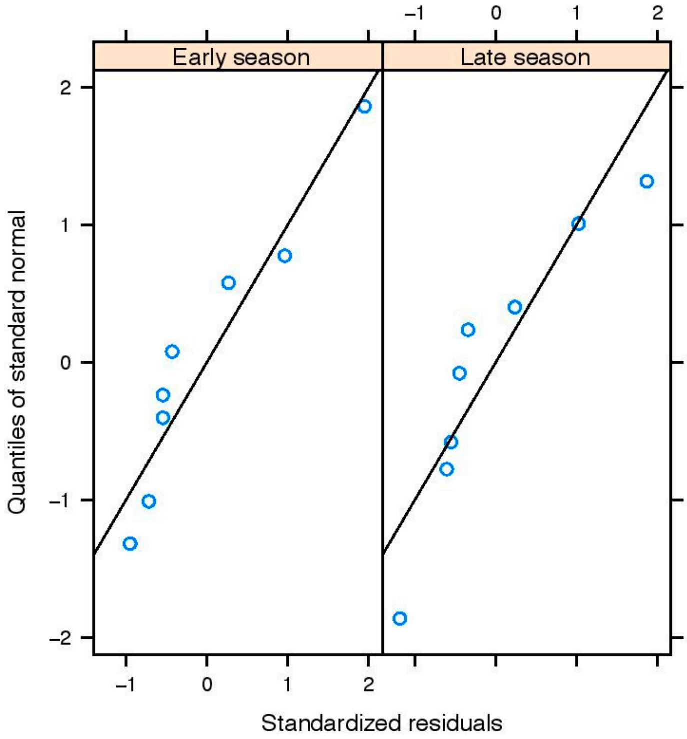

Figure S1. Residual plot from GLS model of nest numbers per season

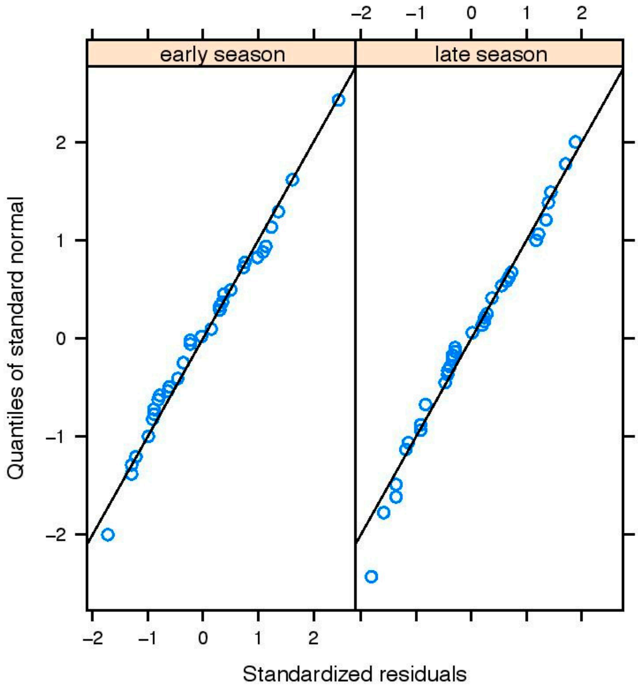

Figure S2. Residual plot from GLS model of colony duration data

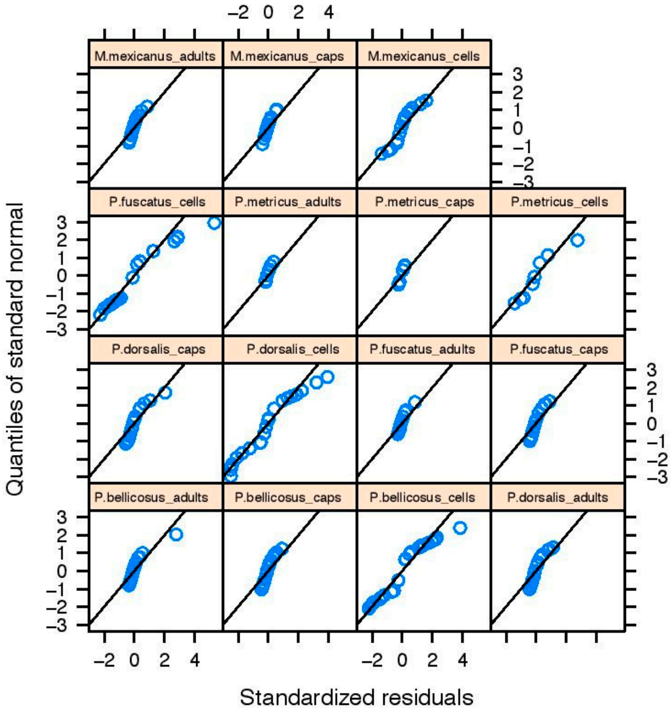

Figure S3. Residual plots from GLS model of cells, adults, and caps for each species

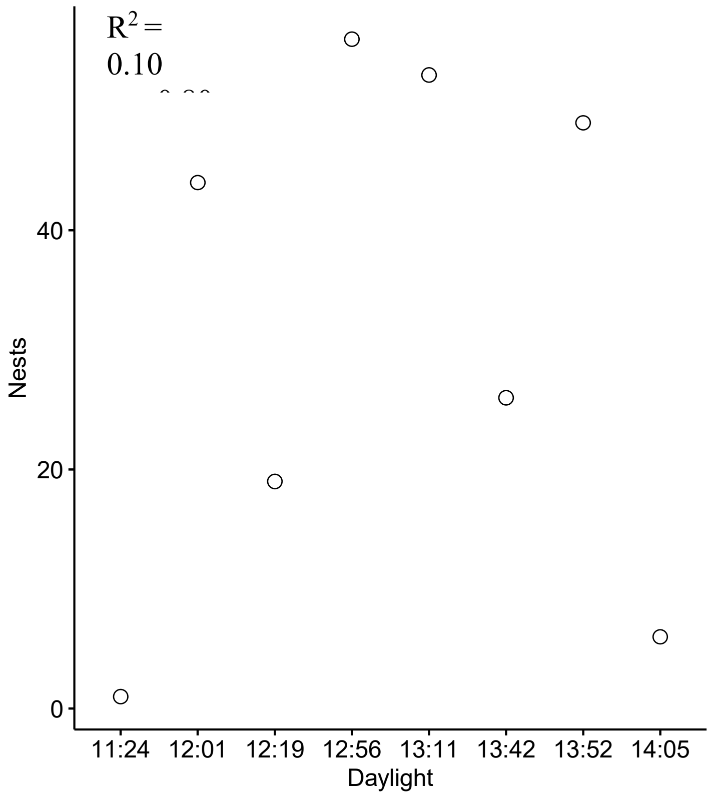

Figure S4. Correlation between number of polistine nests and day length (hrs:mins)

Table S1. Pairwise comparisons of nest duration by species in polistine wasps during spring and the late season. Significant values (p<0.05) in bold.

Season	contrast	Est.	SE	df	t-ratio	p-value
Spring	*P. bellicosus - P. dorsalis*	-14.4	19.5	28	-0.74	0.88
	P. bellicosus - P. metricus	-30.6	21.6	28	-1.42	0.50
	P. bellicosus - M. mexicanus	-56	17.8	28	-3.15	**0.02**
	P. dorsalis - P. metricus	-16.2	19.5	28	-0.83	0.84
	P. dorsalis - M. mexicanus	-41.6	15.2	28	-2.75	**0.05**
	P. metricus - M. mexicanus	-25.4	17.8	28	-1.43	0.49
Late season	*P. bellicosus - P. dorsalis*	-14.28	9.36	29	-1.53	0.55
	P. bellicosus - P. fuscatus	3.13	9.7	29	0.32	1.00
	P. bellicosus - P. metricus	-5.12	16.39	29	-0.31	1.00
	P. bellicosus - M. mexicanus	-32.62	16.39	29	-1.99	0.30
	P. dorsalis - P. fuscatus	17.42	10.49	29	1.66	0.47
	P. dorsalis - P. metricus	9.17	16.87	29	0.54	0.98
	P. dorsalis - M. mexicanus	-18.33	16.87	29	-1.09	0.81
	P. fuscatus - P. metricus	-8.25	17.06	29	-0.48	0.99
	P. fuscatus - M. mexicanus	-35.75	17.06	29	-2.10	0.25
	P. metricus - M. mexicanus	-27.5	21.58	29	-1.27	0.71

Table S2. Model results of nesting parameters in Polistine wasps: Peak number of adults, caps and cells for each of five species. Significant values (p<0.05) in bold.

Species	Form	Est.	Std. Error	t-value	p-value
Polistes bellicosus	Adults	10.88	2.26	4.81	**0.00**
	Caps	16.62	3.71	4.48	**0.00**
	Cells	94.84	13.86	6.84	**0.00**
Polistes dorsalis	Adults	5.83	6.80	0.86	0.40
	Caps	13.30	8.90	1.49	0.15
	Cells	55.14	24.27	2.27	**0.03**
Polistes fuscatus	Adults	5.60	4.19	1.34	0.20
	Caps	13.91	5.62	2.48	**0.02**
	Cells	84.79	28.04	3.02	**0.01**
Polistes metricus	Adults	2.83	5.04	0.56	0.59
	Caps	2.17	7.50	0.29	0.78
	Cells	39.67	29.22	1.36	0.22
Mischocyttarus mexicanus	Adults	2.17	2.93	0.74	0.47
	Caps	32.73	6.20	5.28	**0.00**
	Cells	32.73	6.20	5.28	**0.00**

Table S3. Model results of the effects of season, nesting strategy and the interaction between the two pooled for all Polistine wasp species recorded. Result for season against the reference category *Spring*, nesting strategy against the reference category *Single Foundress.* Significant values (p<0.05) in bold.

	Est.	Std. Error	z value	Pr(>|z|)
Season_late	-0.59	0.67	-0.88	0.38
Queens_multiple	-1.23	0.66	-1.86	0.06
Queens_unknown	-0.45	0.64	-0.71	0.48
Season_late:Queens_multiple	2.71	0.96	2.83	**0.00**
Season_late:Queens_unknown	-2.21	1.07	-2.06	**0.04**

Table S4. Data from Polistine nests initiated in the spring season 2016.

**Nest #**	**Species**	**Discovery**	**Foundress**	**Failed before July?**	**Duration (days)**	**Cells**	**Adults**	**Caps**
a1	*P. annularis*	6/04/2016	single	yes	--	--	--	--
b1	*P. bellicosus*	15/03/2016	single	yes	--	--	--	--
b2	*P. bellicosus*	6/04/2016	multiple	yes	--	--	--	--
b3	*P. bellicosus*	12/04/2016	multiple	no	131	171	20	35
b4	*P. bellicosus*	14/04/2016	single	no	--	178	15	33
b5	*P. bellicosus*	25/04/2016	multiple	yes	--	--	--	--
b6	*P. bellicosus*	25/04/2016	multiple	no	--	--	--	--
b7	*P. bellicosus*	25/04/2016	single	no	--	--	--	--
b8	*P. bellicosus*	25/04/2016	single	no	115	136	13	21

b9	*P. bellicosus*	25/04/2016	single	no	--	--	--	--
b10	*P. bellicosus*	2/05/2016	unkn	yes	--	--	--	--
b11	*P. bellicosus*	19/05/2016	single	yes	--	--	--	--
b12	*P. bellicosus*	31/05/2016	multiple	no	130	103	14	15
b13	*P. bellicosus*	31/05/2016	multiple	no	142	169	30	39
b14	*P. bellicosus*	31/05/2016	unkn	no	--	239	27	25
b15	*P. bellicosus*	13/06/2016	unkn	no	--	--	--	--
b16	*P. bellicosus*	17/06/2016	multiple	yes	--	--	--	--
b17	*P. bellicosus*	17/06/2016	unkn	no	--	149	21	52
b18	*P. bellicosus*	17/06/2016	unkn	no	--	118	16	22
b19	*P. bellicosus*	17/06/2016	unkn	no	--			
b20	*P. bellicosus*	17/06/2016	multiple	no	148	181	17	38
b21	*P. bellicosus*	17/06/2016	unkn	no	--	104	13	15
b22	*P. bellicosus*	17/06/2016	unkn	no	--	144	19	22
b23	*P. bellicosus*	17/06/2016	unkn	no	--	142	18	19
b24	*P. bellicosus*	17/06/2016	unkn	no	--	--	--	--
b25	*P. bellicosus*	17/06/2016	unkn	no	--	159	37	33
b26	*P. bellicosus*	20/06/2016	unkn	no	--	--	--	--
b27	*P. bellicosus*	20/06/2016	unkn	no	--	--	--	--
b28	*P. bellicosus*	20/06/2016	unkn	no	--	--	--	--
b29	*P. bellicosus*	20/06/2016	unkn	no	--	--	--	--
b30	*P. bellicosus*	20/06/2016	unkn	no	--	--	--	--
b31	*P. bellicosus*	20/06/2016	unkn	no	--	--	--	--
b32	*P. bellicosus*	20/06/2016	unkn	no	--	--	--	--
b33	*P. bellicosus*	20/06/2016	unkn	no	--	--	--	--
b34	*P. bellicosus*	20/06/2016	unkn	no	--	--	--	--
b35	*P. bellicosus*	20/06/2016	unkn	no	--	--	--	--
b36	*P. bellicosus*	20/06/2016	unkn	no	--	--	--	--
d1	*P. dorsalis*	15/03/2016	single	yes	--	--	--	--
d2	*P. dorsalis*	25/03/2016	multiple	no	131	189	15	28
d3	*P. dorsalis*	25/03/2016	multiple	no	196	175	34	29
d4	*P. dorsalis*	25/03/2016	single	no	--	--	--	--
d5	*P. dorsalis*	31/03/2016	multiple	no	134	54	4	9
d6	*P. dorsalis*	31/03/2016	multiple	yes	--	--	--	--
d7	*P. dorsalis*	6/04/2016	single	yes	--	--	--	--
d8	*P. dorsalis*	7/04/2016	single	no	--	--	--	--
d9	*P. dorsalis*	7/04/2016	multiple	yes	--	--	--	--
d10	*P. dorsalis*	8/04/2016	multiple	yes	--	--	--	--
d11	*P. dorsalis*	8/04/2016	multiple	no	209	238	61	66
d12	*P. dorsalis*	14/04/2016	multiple	no	--	--	--	--
d13	*P. dorsalis*	21/04/2016	multiple	no	98	55	4	6
d14	*P. dorsalis*	21/04/2016	single	yes	--	--	--	--
d15	*P. dorsalis*	3/05/2016	multiple	no	--	--	--	--
d16	*P. dorsalis*	18/05/2016	multiple	no	--	167	10	41
d17	*P. dorsalis*	20/05/2016	multiple	yes	--	--	--	--

d18	*P. dorsalis*	20/05/2016	multiple	no	--	--	--	--
d19	*P. dorsalis*	20/05/2016	multiple	no	157	176	21	25
d20	*P. dorsalis*	20/05/2016	multiple	no	115	120	12	26
d21	*P. dorsalis*	20/05/2016	unkn	no	--	--	--	--
d22	*P. dorsalis*	25/05/2016	single	yes	--	--	--	--
d23	*P. dorsalis*	25/05/2016	single	yes	--	--	--	--
d24	*P. dorsalis*	1/06/2016	unkn	no	--	109	10	21
d25	*P. dorsalis*	14/06/2016	single	yes	--	--	--	--
d26	*P. dorsalis*	14/06/2016	single	yes	--	--	--	--
d27	*P. dorsalis*	14/06/2016	unkn	no	141	187	29	28
d28	*P. dorsalis*	14/06/2016	unkn	no	--	202	48	103
d29	*P. dorsalis*	14/06/2016	multiple	no	--	--	--	--
d30	*P. dorsalis*	14/06/2016	unkn	no	--	--	--	--
e1	*P. exclamans*	25/03/2016	single	yes	--	--	--	--
e2	*P. exclamans*	5/04/2016	single	yes	--	--	--	--
e3	*P. exclamans*	5/04/2016	single	yes	--	--	--	--
e4	*P. exclamans*	8/04/2016	single	yes	--	--	--	--
e5	*P. exclamans*	12/04/2016	single	yes	--	--	--	--
e6	*P. exclamans*	12/04/2016	single	yes	--	--	--	--
e7	*P. exclamans*	19/05/2016	single	no	--	--	--	--
e8	*P. exclamans*	8/06/2016	unkn	no	--	--	--	--
e9	*P. exclamans*	16/06/2016	unkn	no	--	--	--	--
e10	*P. exclamans*	16/06/2016	unkn	no	--	--	--	--
e11	*P. exclamans*	17/06/2016	unkn	no	--	--	--	--
e12	*P. exclamans*	17/06/2016	unkn	no	--	--	--	--
f1	*P. fuscatus*	3/03/2016	multiple	no	--	46	7	6
f2	*P. fuscatus*	15/03/2016	multiple	yes	--	--	--	--
f3	*P. fuscatus*	31/03/2016	single	no	--	--	--	--
f4	*P. fuscatus*	31/03/2016	single	yes	--	--	--	--
f5	*P. fuscatus*	6/04/2016	single	yes	--	--	--	--
f6	*P. fuscatus*	6/04/2016	multiple	no	--	--	--	--
f7	*P. fuscatus*	6/04/2016	single	yes	--	--	--	--
f8	*P. fuscatus*	6/04/2016	unkn	no	--	291	45	32
f9	*P. fuscatus*	6/04/2016	unkn	no	--	60	8	15
f10	*P. fuscatus*	14/06/2016	unkn	no	--	192	13	16
f11	*P. fuscatus*	14/06/2016	unkn	no	--	140	12	32
f12	*P. fuscatus*	14/06/2016	unkn	no	--	200	23	41
f13	*P. fuscatus*	14/06/2016	unkn	no	--	171	22	51
f14	*P. fuscatus*	14/06/2016	unkn	no	--	100	10	24
f15	*P. fuscatus*	14/06/2016	unkn	no	--	89	16	18
f16	*P. fuscatus*	14/06/2016	unkn	no	--	51	10	13
f17	*P. fuscatus*	14/06/2016	unkn	no	--	--	--	--
m1	*P. metricus*	25/03/2016	single	yes	--	--	--	--
m2	*P. metricus*	25/03/2016	single	yes	--	--	--	--
m3	*P. metricus*	31/03/2016	single	no	157	27	3	4

m4	*P. metricus*	5/04/2016	single	no	205	34	3	6
m5	*P. metricus*	7/04/2016	single	no	178	167	22	18
m6	*P. metricus*	13/04/2016	single	yes	--	--	--	--
m7	*P. metricus*	20/04/2016	single	no	--	--	--	--
m8	*P. metricus*	25/04/2016	single	no	152	61	15	14
m9	*P. metricus*	3/05/2016	single	yes	--	--	--	--
m10	*P. metricus*	3/05/2016	single	yes	--	--	--	--
m11	*P. metricus*	3/05/2016	single	yes	--	--	--	--
m12	*P. metricus*	8/06/2016	unkn	yes	127	74	10	19
m13	*P. metricus*	22/06/2016	single	yes	--	--	--	--
m14	*P. metricus*	22/06/2016	unkn	no	--	94	6	18
mx1	*M. mexicanus*	25/03/2016	multiple	no	--	121	26	41
mx2	*M. mexicanus*	25/03/2016	single	yes	--	--	--	--
mx3	*M. mexicanus*	25/03/2016	single	yes	--	--	--	--
mx4	*M. mexicanus*	25/03/2016	single	yes	--	--	--	--
mx5	*M. mexicanus*	25/03/2016	single	yes	--	--	--	--
mx6	*M. mexicanus*	25/03/2016	single	yes	--	--	--	--
mx7	*M. mexicanus*	25/03/2016	single	yes	--	--	--	--
mx8	*M. mexicanus*	25/03/2016	single	yes	--	--	--	--
mx9	*M. mexicanus*	25/03/2016	single	yes	--	--	--	--
mx10	*M. mexicanus*	25/03/2016	single	yes	--	--	--	--
mx11	*M. mexicanus*	25/03/2016	single	yes	--	--	--	--
mx12	*M. mexicanus*	25/03/2016	single	yes	--	--	--	--
mx13	*M. mexicanus*	25/03/2016	single	yes	--	--	--	--
mx14	*M. mexicanus*	25/03/2016	single	yes	--	--	--	--
mx15	*M. mexicanus*	1/04/2016	single	yes	--	--	--	--
mx16	*M. mexicanus*	1/04/2016	multiple	no	263	83	13	23
mx17	*M. mexicanus*	6/04/2016	multiple	no	230	81	13	20
mx18	*M. mexicanus*	6/04/2016	single	no	220	79	21	23
mx19	*M. mexicanus*	6/04/2016	multiple	no	--	25	4	6
mx20	*M. mexicanus*	6/04/2016	multiple	no	215	62	15	17
mx21	*M. mexicanus*	6/04/2016	multiple	no	135	94	13	21
mx22	*M. mexicanus*	6/04/2016	single	yes	--	--	--	--
mx23	*M. mexicanus*	8/04/2016	single	yes	--	--	--	--
mx24	*M. mexicanus*	8/04/2016	single	yes	--	--	--	--
mx25	*M. mexicanus*	13/04/2016	single	yes	--	--	--	--
mx26	*M. mexicanus*	13/04/2016	single	yes	--	--	--	--
mx27	*M. mexicanus*	25/04/2016	unkn	yes	--	--	--	--
mx28	*M. mexicanus*	25/04/2016	single	yes	--	--	--	--
mx29	*M. mexicanus*	25/04/2016	multiple	yes	--	--	--	--
mx30	*M. mexicanus*	25/04/2016	multiple	yes	--	--	--	--
mx31	*M. mexicanus*	25/04/2016	unkn	yes	--	--	--	--
mx32	*M. mexicanus*	4/05/2016	multiple	no	178	69	19	16
mx33	*M. mexicanus*	10/05/2016	unkn	no	195	78	11	14
mx34	*M. mexicanus*	11/05/2016	single	no	180	80	18	27

mx35	*M. mexicanus*	12/05/2016	multiple	yes	--	--	--	--
mx36	*M. mexicanus*	18/05/2016	single	no	--	--	--	--
mx37	*M. mexicanus*	18/05/2016	single	yes	--	--	--	--
mx38	*M. mexicanus*	20/05/2016	unkn	no	--	82	10	19
mx39	*M. mexicanus*	25/05/2016	single	no	186	73	6	19
mx40	*M. mexicanus*	25/05/2016	multiple	no	181	64	22	16
mx41	*M. mexicanus*	1/06/2016	single	yes	--	--	--	--
mx42	*M. mexicanus*	8/06/2016	unkn	no	211	100	25	15
mx43	*M. mexicanus*	8/06/2016	multiple	no	172	40	10	13
mx44	*M. mexicanus*	15/06/2016	single	yes	--	--	--	--
mx45	*M. mexicanus*	15/06/2016	multiple	no	118	46	10	10
mx46	*M. mexicanus*	17/06/2016	single	no	--	83	13	15
mx47	*M. mexicanus*	21/06/2016	unkn	no	--	--	--	--
mx48	*M. mexicanus*	21/06/2016	unkn	no	--	--	--	--
mx49	*M. mexicanus*	21/06/2016	multiple	no	165	135	49	40
mx50	*M. mexicanus*	21/06/2016	unkn	no	--	--	--	--
mx51	*M. mexicanus*	21/06/2016	unkn	no	--	90	17	41
mx52	*M. mexicanus*	21/06/2016	unkn	no	--	123	35	24
mx53	*M. mexicanus*	21/06/2016	unkn	no	--	--	--	--
mx54	*M. mexicanus*	21/06/2016	unkn	no	--	--	--	--
u1	undet.	15/03/2016	single	yes	--	--	--	--
u2	undet.	15/03/2016	single	yes	--	--	--	--
u3	undet.	25/03/2016	single	yes	--	--	--	--
u4	undet.	25/03/2016	single	yes	--	--	--	--
u5	undet.	31/03/2016	multiple	yes	--	--	--	--
u6	undet.	6/04/2016	single	yes	--	--	--	--
u7	undet.	12/04/2016	single	yes	--	--	--	--
u8	undet.	13/04/2016	single	no	--	--	--	--
u9	undet.	14/04/2016	single	yes	--	--	--	--
u10	undet.	21/04/2016	single	yes	--	--	--	--
u11	undet.	21/04/2016	unkn	yes	--	--	--	--
u12	undet.	21/04/2016	unkn	yes	--	--	--	--

Table S5: Data from Polistine nests initiated in late season 2016.

**Nest#**	**Species**	**Discovery**	**Foundress**	**Duration (days)**	**Cells**	**Adults**	**Caps**
b37	*P. bellicosus*	28/06/2016	multiple	--	--	--	--
b38	*P. bellicosus*	19/07/2016	multiple	--	--	--	--
b39	*P. bellicosus*	19/07/2016	single	--	--	--	--
b40	*P. bellicosus*	20/07/2016	multiple	94	74	7	10
b41	*P. bellicosus*	26/07/2016	multiple	--	--	--	--
b42	*P. bellicosus*	28/07/2016	multiple	93	112	21	30
b43	*P. bellicosus*	2/08/2016	multiple	--	--	--	--
b44	*P. bellicosus*	2/08/2016	multiple	--	--	--	--
b45	*P. bellicosus*	2/08/2016	single	--	--	--	--

b46	*P. bellicosus*	2/08/2016	single	--	--	--	--
b47	*P. bellicosus*	2/08/2016	multiple	--	--	--	--
b48	*P. bellicosus*	2/08/2016	single	--	--	--	--
b49	*P. bellicosus*	2/08/2016	multiple	--	--	--	--
b50	*P. bellicosus*	9/08/2016	multiple	69	53	5	4
b51	*P. bellicosus*	9/08/2016	multiple	67	44	7	7
b52	*P. bellicosus*	9/08/2016	multiple	115	77	8	7
b53	*P. bellicosus*	9/08/2016	multiple	--	30	5	6
b54	*P. bellicosus*	9/08/2016	multiple	--	--	--	--
b55	*P. bellicosus*	9/08/2016	single	--	--	--	--
b56	*P. bellicosus*	9/08/2016	single	--	--	--	--
b57	*P. bellicosus*	9/08/2016	single	78	20	5	3
b58	*P. bellicosus*	9/08/2016	single	120	87	15	14
b59	*P. bellicosus*	9/08/2016	single	--	--	--	--
b60	*P. bellicosus*	10/08/2016	multiple	--	171	12	33
b61	*P. bellicosus*	10/08/2016	multiple	119	49	10	15
b62	*P. bellicosus*	10/08/2016	multiple	--	--	--	--
b63	*P. bellicosus*	10/08/2016	multiple	93	49	11	10
b64	*P. bellicosus*	11/08/2016	multiple	--	--	--	--
b65	*P. bellicosus*	9/08/2016	single	--	--	--	--
b66	*P. bellicosus*	16/08/2016	multiple	--	--	--	--
b67	*P. bellicosus*	16/08/2016	multiple	--	55	10	20
b68	*P. bellicosus*	16/08/2016	single	--	--	--	--
b69	*P. bellicosus*	16/08/2016	single	--	--	--	--
b70	*P. bellicosus*	16/08/2016	multiple	--	--	--	--
b71	*P. bellicosus*	16/08/2016	multiple	--	30	11	6
b72	*P. bellicosus*	16/08/2016	single	--	--	--	--
b73	*P. bellicosus*	16/08/2016	multiple	--	--	--	--
b74	*P. bellicosus*	16/08/2016	multiple	67	72	11	15
b75	*P. bellicosus*	22/08/2016	multiple	--	--	--	--
b76	*P. bellicosus*	22/08/2016	single	--	--	--	--
b77	*P. bellicosus*	22/08/2016	multiple	52	23	5	8
b78	*P. bellicosus*	30/08/2016	single	--	--	--	--
b79	*P. bellicosus*	30/08/2016	multiple	--	--	--	--
b80	*P. bellicosus*	30/08/2016	multiple	--	--	--	--
b81	*P. bellicosus*	30/08/2016	multiple	--	--	--	--
b82	*P. bellicosus*	6/09/2016	multiple	--	--	--	--
b83	*P. bellicosus*	6/09/2016	multiple	--	--	--	--
b84	*P. bellicosus*	20/09/2016	multiple	47	27	5	8
b85	*P. bellicosus*	20/09/2016	single	--	--	--	--
b86	*P. bellicosus*	20/09/2016	multiple	57	21	7	4
d31	*P. dorsalis*	6/07/2016	multiple	--	--	--	--
d32	*P. dorsalis*	6/07/2016	multiple	--	--	--	--
d33	*P. dorsalis*	8/07/2016	multiple	103	114	23	13
d34	*P. dorsalis*	8/07/2016	multiple	--	132	9	40

d35	*P. dorsalis*	13/07/2016	multiple	--	--	--	--
d36	*P. dorsalis*	13/07/2016	single	--	--	--	--
d37	*P. dorsalis*	13/07/2016	multiple	--	--	--	--
d38	*P. dorsalis*	20/07/2016	multiple	118	102	16	28
d39	*P. dorsalis*	20/07/2016	multiple	--	120	7	21
d40	*P. dorsalis*	20/07/2016	multiple	104	101	4	18
d41	*P. dorsalis*	28/07/2016	multiple	--	151	13	21
d42	*P. dorsalis*	9/08/2016	multiple	126	253	46	49
d43	*P. dorsalis*	10/08/2016	multiple	78	35	5	9
d44	*P. dorsalis*	10/08/2016	multiple	--	--	--	--
d45	*P. dorsalis*	10/08/2016	multiple	114	34	16	12
d46	*P. dorsalis*	10/08/2016	multiple	--	--	--	--
d47	*P. dorsalis*	10/08/2016	multiple	--	--	--	--
d48	*P. dorsalis*	17/08/2016	single	88	47	22	18
d49	*P. dorsalis*	25/08/2016	multiple	77	119	49	29
d50	*P. dorsalis*	25/08/2016	multiple	--	--	--	--
d51	*P. dorsalis*	2/09/2016	multiple	--	37	10	8
d52	*P. dorsalis*	9/09/2016	multiple	--	77	5	18
d53	*P. dorsalis*	9/09/2016	multiple	--	--	--	--
d54	*P. dorsalis*	9/09/2016	multiple	--	--	--	--
d55	*P. dorsalis*	9/09/2016	multiple	--	--	--	--
d56	*P. dorsalis*	22/09/2016	multiple	62	34	9	16
d57	*P. dorsalis*	16/09/2016	unkn	--	--	--	--
d58	*P. dorsalis*	16/09/2016	multiple	--	--	--	--
d59	*P. dorsalis*	30/09/2016	multiple	--	--	--	--
d60	*P. dorsalis*	4/10/2016	unkn	--	--	--	--
d61	*P. dorsalis*	6/10/2016	multiple	--	--	--	--
e13	*P. exclamans*	26/07/2016	multiple	--	--	--	--
e14	*P. exclamans*	9/08/2016	multiple	--	--	--	--
e15	*P. exclamans*	16/08/2016	unkn	--	--	--	--
f18	*P. fuscatus*	6/07/2016	multiple	61	58	8	10
f19	*P. fuscatus*	6/07/2016	multiple	80	43	6	5
f20	*P. fuscatus*	6/07/2016	multiple	--	--	--	--
f21	*P. fuscatus*	20/07/2016	multiple	--	--	--	--
f22	*P. fuscatus*	26/07/2016	single	81	36	5	6
f23	*P. fuscatus*	28/07/2016	multiple	102	106	22	37
f24	*P. fuscatus*	28/07/2016	single	80	16	13	5
f25	*P. fuscatus*	3/08/2016	multiple	92	46	9	7
f26	*P. fuscatus*	3/08/2016	multiple	--	106	20	12
f27	*P. fuscatus*	10/08/2016	single	81	25	12	13
f28	*P. fuscatus*	10/08/2016	multiple	--	--	--	--
f29	*P. fuscatus*	16/08/2016	multiple	--	--	--	--
f30	*P. fuscatus*	17/08/2016	multiple	57	33	4	3
f31	*P. fuscatus*	17/08/2016	multiple	--	--	--	--
f32	*P. fuscatus*	22/08/2016	unkn	--	--	--	--

f33	*P. fuscatus*	9/09/2016	multiple	--	--	--	--
f34	*P. fuscatus*	4/10/2016	single	--	--	--	--
f35	*P. fuscatus*	6/10/2016	multiple	--	--	--	--
f36	*P. fuscatus*	11/10/2016	single	--	--	--	--
m15	*P. metricus*	27/06/2016	single	--	--	--	--
m16	*P. metricus*	5/07/2016	single	--	--	--	--
m17	*P. metricus*	20/07/2016	multiple	96	57	11	18
m18	*P. metricus*	26/07/2016	single	--	--	--	--
m19	*P. metricus*	26/07/2016	multiple	79	16	3	4
m20	*P. metricus*	28/07/2016	single	--	--	--	--
m21	*P. metricus*	2/08/2016	multiple	--	--	--	--
m22	*P. metricus*	2/08/2016	single	--	--	--	--
mx55	*M. mexicanus*	13/07/2016	single	--	--	--	--
mx56	*M. mexicanus*	20/07/2016	single	--	--	--	--
mx57	*M. mexicanus*	9/08/2016	single	130	50	12	17
mx58	*M. mexicanus*	10/08/2016	multiple	--	45	18	17
mx59	*M. mexicanus*	16/08/2016	multiple	100	48	16	17
mx60	*M. mexicanus*	16/09/2016	single	--	--	--	--
u13	undet.	13/07/2016	single	--	--	--	--
u14	undet.	15/07/2016	single	--	--	--	--
u15	undet.	26/07/2016	single	--	--	--	--
u16	undet.	2/08/2016	single	--	--	--	--
u17	undet.	10/08/2016	multiple	--	--	--	--
u18	undet.	16/08/2016	single	--	--	--	--
u19	undet.	22/09/2016	single	--	--	--	--
